# Evaluating user perceptions and usability of an AI-powered smartphone application for at-home dental plaque screening

**DOI:** 10.1038/s41415-025-8502-0

**Published:** 2025-07-11

**Authors:** Dania Al-Zubaidy, Nicola Innes, Jennifer Galloway, Waraf Al-Yaseen

**Affiliations:** Cardiff University School of Dentistry, University Hospital Wales, Heath Park, Cardiff, CF14 4XY, United Kingdom

## Abstract

**Introduction** Artificial intelligence (AI)-powered mobile applications (apps) like TestMyTeeth offer potential for at-home dental plaque screening.

**Objective** To evaluate usability and user perceptions of the TestMyTeeth app for remote plaque detection.

**Methods** A cross-sectional study with adult participants recruited using convenience sampling. Participants used the app and completed an online questionnaire including the system usability scale (SUS) and user experience items. Descriptive statistics summarised demographics and responses. SUS scores and non-parametric tests assessed usability differences across age, education level and sex.

**Results** For 132 participants recruited (75% female; 90% under 44 years-old), the mean SUS score was 56.2 (standard deviation ± 13.5; range: 10-85), indicating ‘marginal' acceptability. Additionally, 54.6% were interested in frequent use, 41.6% found the app easy to use and 50% needed technical support. Participants found significant challenges capturing intra-oral photographs, especially for posterior teeth. No significant SUS score differences were found for age (*p* = 0.951), education level (*p* = 0.569), or sex (*p* = 0.154). Qualitative feedback highlighted benefits of self-monitoring but concerns about app complexity and accuracy without professional guidance.

**Conclusion** The app shows potential for empowering at-home dental plaque screening but faces usability challenges. Improvements in user interface and photo-capture functionality would enhance usability.

**Supplementary Information:**

Zusatzmaterial online: Zu diesem Beitrag sind unter 10.1038/s41415-025-8502-0 für autorisierte Leser zusätzliche Dateien abrufbar.

## Key points

- The TestMyTeeth app had marginal acceptability for usability, with participants experiencing difficulties using it effectively and anticipating needing technical support.
- Around half of participants were interested in incorporating the app into their oral care routines.
- Participants reported significant challenges in taking clear intra-oral photographs, especially of the back teeth, which is necessary for accurate dental plaque screening.

**Supplementary Information:**

Zusatzmaterial online: Zu diesem Beitrag sind unter 10.1038/s41415-025-8502-0 für autorisierte Leser zusätzliche Dateien abrufbar.

## Introduction

The COVID-19 pandemic accelerated the adoption of digital health solutions, revealing the potential for remote healthcare delivery.^1^ In dentistry, remote dental monitoring has emerged as a promising innovation aimed at improving access to care, reducing the burden of in-person visits and addressing the unique needs of individuals with special health requirements, such as those with autism.^2^ Technologies like intra-oral photography and artificial intelligence (AI)-driven tools enable remote diagnosis, continuous monitoring and tailored treatment plans, transforming dental care delivery.^3^

TestMyTeeth is an example of an AI-powered smartphone application (app) designed for at-home dental plaque screening. Using a smartphone's camera and advanced image analysis algorithms, TestMyTeeth enables users to capture intra-oral photographs and receive real-time feedback on plaque accumulation. This self-monitoring tool aims to empower individuals to take proactive steps in their oral health maintenance, potentially reducing the burden on dental professionals and enhancing preventive care.^4^

Usability is a critical factor for the successful adoption of mobile health (mHealth) applications like TestMyTeeth. Assessing usability involves evaluating how effectively end-users can interact with an app to achieve their goals - in this case, accurate self-assessment of dental plaque. Tools such as the system usability scale (SUS), heuristic evaluations and user interviews are commonly employed to measure usability and user experience.^5^^,^^6^ High usability is associated with increased user satisfaction, engagement and adherence, which are essential for the app's effectiveness in promoting oral health.^6^^,^^7^

While the literature on mHealth apps and AI in dentistry is growing,^8^ there is a paucity of research focusing on end-user perceptions of these technologies, particularly in the context of dental plaque assessment.^5^ This study aims to address this gap by investigating the usability and user experience of AI-driven dental plaque monitoring apps among diverse populations.

## Aims

To evaluate the usability and user perceptions of the TestMyTeeth AI-powered smartphone app for remote dental plaque detection among adult users.

## Objectives

1. To measure the usability of the TestMyTeeth app and its acceptability across different demographic groups (age, education level, sex)
2. To identify usability challenges experienced by users, focusing on the difficulties in capturing clear intra-oral photographs of back teeth, essential for accurate dental plaque screening
3. To examine user perceptions regarding the effectiveness of the TestMyTeeth app in promoting oral health self-monitoring, including perceived benefits, limitations and the willingness to incorporate it into daily dental care routines.

## Materials and methods

### Study design and outcomes

This study employed a cross-sectional, prospective, survey-based design to evaluate the usability and user experience of the hybrid paediatric dental care model's digital app. The primary outcome was usability, measured using the SUS score. Secondary outcomes included the end-user experience, which encompassed satisfaction and ease of navigation, assessed through additional questionnaire items. Furthermore, qualitative feedback was collected to explore user experiences in depth and identify areas for improvement. Another secondary outcome examined the association between participants' perceptions of app usability and their demographic backgrounds.

### Ethical considerations

The study received a favourable opinion from the School of Dentistry Research Ethics Committee at Cardiff University, as it was deemed to be of low risk (ID no. 2334a, date: 14.11.2023). Data, such as names or facial photographs, were not collected, ensuring participants' anonymity. Participants were provided with a detailed information sheet, outlining the study's aims, and were required to give informed consent before uploading intra-oral photographs. The right to withdraw from the study at any point was ensured, and data collection strictly adhered to ethical guidelines regarding confidentiality and data protection.

### Data collection questionnaire tool

The questionnaire used in this study was adapted from previous research on AI-powered smartphone apps for dental caries screening, validated by Al-Jallad *et al.*^9^ It included the SUS score, a widely recognised tool for measuring usability.^6^ The questionnaire consisted of ten items related to ease of use, functionality and user satisfaction, with participants rating their agreement on a five-point Likert scale. The questionnaire also included demographic questions and open-ended items for qualitative feedback, such as experiences with the app and suggestions for improvement. The questionnaire was administered through Microsoft Forms (Microsoft, Richmond, USA) (see online Supplementary Information File 1).

### Selection of participants

Participants aged 16 years and older who had access to a mobile phone with a functioning camera and provided informed consent were included in the study. Participants aged 16 years and above were chosen due to being of the legal age to provide informed consent to participate in research. Individuals who did not provide explicit consent or lacked access to a suitable mobile phone were excluded. To enhance diversity, efforts were made to balance representation across different age groups. Based on insights from a scoping review of similar studies assessing mHealth app usability,^6^ the study aimed to recruit at least 60-65 participants.

### Recruitment process

Participants were recruited through convenience purposive sampling via Cardiff University's internal communication platforms (e.g., Microsoft Yammer) and the research team's networks. While convenience sampling may favour individuals who are more engaged with research or comfortable with technology, we attempted to reduce this bias by incorporating purposive and snowball sampling. Participants were encouraged to share the study with colleagues, helping to reach individuals with varying levels of digital literacy and professional backgrounds. Recruitment materials, including participant information sheets and consent forms, were distributed electronically (see online Supplementary Information File 2). The recruitment period spanned six months (November 2023 to May 2024) to allow for broader outreach.

## Data collection

Participants were directed to download the TestMyTeeth app, with a link to the validated questionnaire embedded within the application (online Supplementary Information Appendix 1). The questionnaire began with consent statements and included detailed instructions on app usage. Participants were provided with disclosing tablets upon request to aid in capturing accurate intra-oral images. Guidance was provided at each step to ensure that participants could complete the questionnaire and app tasks successfully. All the questionnaire questions were made as mandatory to fill to avoid missing data. Data were stored securely and anonymously via Microsoft Forms.

### Analysis and outcomes

#### Demographic overview

Descriptive statistics were used to summarise participant demographics, including age, sex and socioeconomic status.

#### SUS score

The SUS scores were calculated by summing participants' responses to the ten items, resulting in a composite score for each participant. The SUS scores ranged from 0-100, with higher scores indicating better usability. A median SUS score was calculated for the entire cohort, where a score above 68 was considered above average. Scores between 70-84 suggest good usability, while scores above 85 indicate excellent usability. Scores below 50 typically indicate significant usability challenges. The median score was compared against these benchmarks to assess the relative performance of the TestMyTeeth app.

#### Non-SUS questionnaire responses

Additional quantitative items in the questionnaire (beyond SUS) were analysed to capture broader aspects of user experience, including ease of navigation, satisfaction with the app's visual design and perceived usefulness. These responses were evaluated using descriptive statistics, such as means, medians and frequency distributions.

#### Comparative analysis

To assess whether demographic factors influenced SUS scores, non-parametric statistical tests were employed due to the ordinal nature of the data. The Kruskal-Wallis test was used for comparing SUS scores across multiple independent groups (age categories and education levels), while the Mann-Whitney *U* test was applied for two-group comparisons (sex). A significance level of p <0.05 was considered statistically significant.

#### Qualitative analysis

Open-ended responses were analysed and grouped into themes. The data were coded and grouped into key topic summaries, such as ease of app use, suggestions for improvement and perceived barriers. These topic summaries provided additional context and complemented the quantitative data, offering a deeper understanding of participants' experiences.

## Results

### Participant demographics

A total of 132 participants completed the study, providing a diverse sample for analysis. The majority of participants were female (75.8%; n = 100) and aged between 16-44 years (90.1%; n = 119). Regarding education levels, 58.4% (n = 77) had attained university-level education, with 37.9% (n = 50) holding an undergraduate degree and 20.5% (n = 27) a postgraduate degree. Additionally, 18.2% (n = 24) had completed college or sixth-form qualifications and 13.6% (n = 18) had secondary school qualifications. Prior experience with dental photography using mobile phones was reported by 52.2% (n = 72) of participants before using the TestMyTeeth app. Detailed demographics are presented in Table 1.

**Table 1:** Demographic profile of participants in the study (n = 132)

**Category**	**Count (n)**	**Percentage (%)**
**Age**		
16-24	45	34.1%
25-34	37	28.0%
35-44	37	28.0%
45+	13	9.8%
**Sex**		
Female	100	75.8%
Male	32	24.2%
**Education level**		
Secondary school (GCSEs or equivalent)	18	13.6%
College/Sixth form (A level/BTEC or equivalent)	24	18.2%
University undergraduate level	50	37.9%
University postgraduate level	27	20.5%
Vocational training	13	9.8%
**Previous use of mobile phone for dental photography**		
Yes	72	52.2%
No	66	47.8%

### Usability assessment SUS score

The usability of the TestMyTeeth app was evaluated using the SUS. The median SUS score among participants was 56.2 (standard deviation [SD] ± 13.5; range: 10-85), which falls into the ‘marginal' acceptability category.

For usability, 41.6% (n = 55) found the app easy to use, with 37.9% (n = 50) remaining neutral. Confidence in using the app was expressed by 49.1% (n = 53), yet half of the participants (50%; n = 66) indicated they might need technical support to use the app effectively. Additionally, 51.5% (n = 68) agreed or strongly agreed that the app was unnecessarily complex and 48.5% (n = 64) felt they needed to learn several things before effectively using it. When asked about the likelihood of them using the app frequently, 54.6% (n = 72) of participants agreed or strongly agreed that they would.

**Fig. 1:**
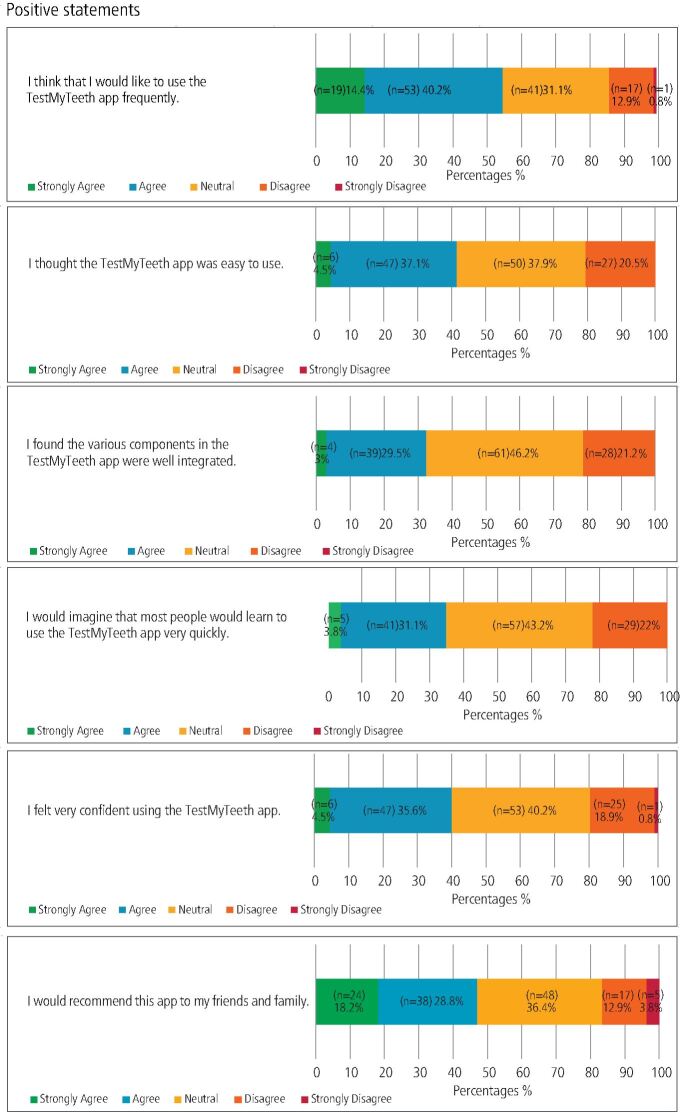
Distribution of responses to SUS items: positive statements

**Fig. 2:**
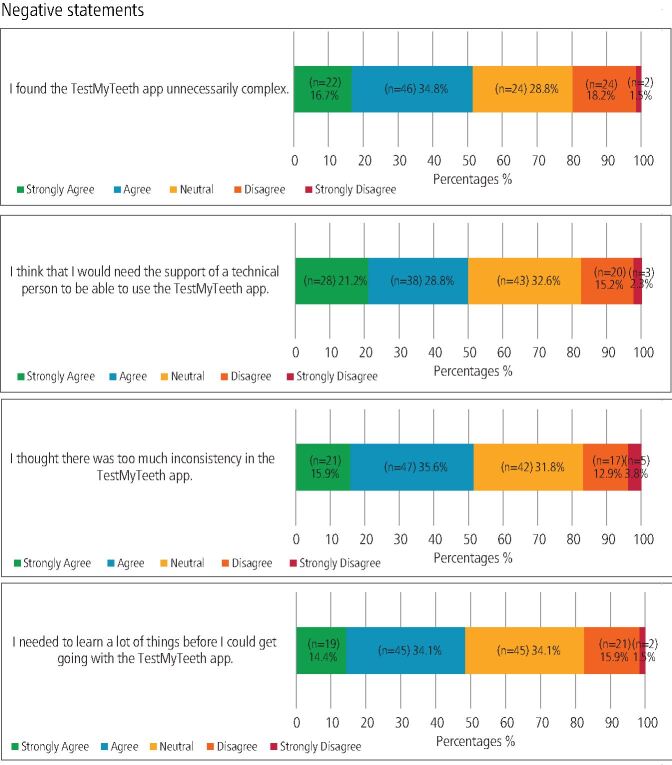
Distribution of responses to SUS items: negative statements

The majority of respondents were positive (18.2% n = 24) or neutral (36.4% n = 48) when asked if they would recommend the app to friends and family. Detailed responses to the SUS items are provided in Figure 1 and Figure 2.

### Statistical analysis of SUS scores

Statistical analysis revealed no evidence of a difference in SUS scores when examined against demographic variables (Table 2). For age groups and education levels, the Kruskal-Wallis test was used, yielding a *p* value of 0.951 and 0.569, respectively. Differences between sexes were also not significant, with the Mann-Whitney *U* test and a *p* value of 0.154.

**Table 2:** Statistical correlation between SUS scores and demographic variables (n = 132)

**Variable**	**Statistic (test)**	**Degrees of freedom**	**p value**
Education level	χ^²^ = 2.015 (Kruskal-Wallis)	4	0.569
Age	χ^²^ = 0.349 (Kruskal-Wallis)	3	0.951
Sex	*U* = 842 (Mann-Whitney *U* test)	N/A	0.154

### Perceived user experience

**Fig. 3:**
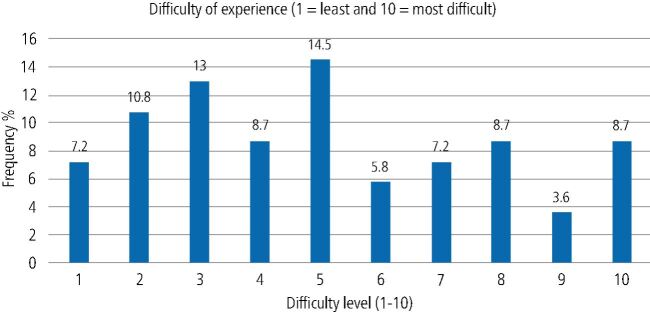
Difficulty of the experience ranked from 1-10 (n = 132)

Participants reported varying levels of difficulty when using the TestMyTeeth app. While some found the app easy to use, a notable proportion (21%) rated the difficulty level between 8-10 on a scale, where ten represents the highest difficulty (Fig. 3).

**Fig. 4:**
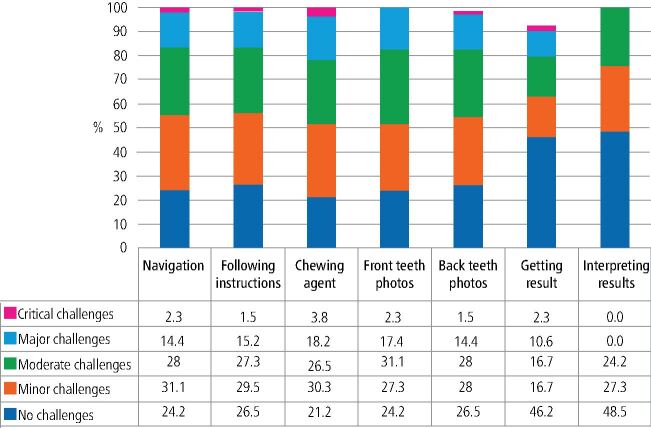
Count and frequency of challenges reported while going through the process of using the app (n = 132)

Specifically, 24.2% (n = 32) reported no challenges with navigation, whereas a combined 45.5% (n = 60) faced minor to moderate difficulties. In terms of following instructions, 43.5% (n = 57) encountered moderate to major difficulties. Capturing intra-oral photographs posed significant challenges. Nearly half of the participants (approximately 50%) reported moderate to major difficulties in taking photos of their front teeth and 42.4% faced similar challenges with their back teeth (Fig. 4).

### Qualitative feedback

#### Views on self-monitoring of oral health

Participants expressed both positive and negative perceptions regarding self-monitoring of oral health using the TestMyTeeth app. On the positive side, many viewed self-monitoring as a valuable tool for early detection of dental issues and for empowering individuals to take responsibility for their own health. For example, one participant stated: ‘it's important to self-monitor because these details will be valuable when a dental problem arises'. Others appreciated the educational aspect, noting that it helps people check their oral health regularly and could reduce unnecessary visits to dental professionals.

Conversely, some participants raised concerns about the accuracy and reliability of self-monitoring without professional input. One participant commented: ‘not everyone is knowledgeable enough…certain health problems may be missed'. Others feared that self-monitoring might lead to unnecessary anxiety or misinterpretation of symptoms, potentially delaying professional treatment. These mixed views highlight the need for balancing user empowerment with accurate guidance and support within the app. Key themes and representative quotes are summarised in Table 3.

**Table 3:** Analysis of participants' views on self-monitoring oral health, categorised into key themes reflecting positive and negative perspectives

**Theme**	**Participant quotes**
Patient empowerment and ownership	‘Gives the power over to the patient to be responsible for their own health' ‘It's important to self-monitor because these details will be valuable when a dental problem arises'
Promoting preventive behaviour	‘A really good way for people to check their oral hygiene regularly' ‘It can reduce waiting times and teach patients how to monitor their dental health'
Insufficient clinical knowledge	‘Not everyone is knowledgeable enough…certain health problems may be missed' ‘Self-monitoring is less of a good idea if the methods you are using are not accurate'
Health anxiety and diagnostic uncertainty	‘Could lead to unnecessary anxiety or misinterpretation of symptoms' ‘Best to get opinion from professional'
Reliability and accuracy concerns	‘Self-monitoring is less of a good idea if the methods you are using are not accurate' ‘Best to get opinion from professional'
Need for professional oversight	‘Best to get opinion from professional' ‘Not everyone is knowledgeable enough…certain health problems may be missed'

#### Technical difficulties and suggestions for improvement

Participants identified several technical challenges and provided suggestions for enhancements. Common technical issues included difficulty in taking clear photos, particularly of the back teeth, and problems with camera focus leading to blurry images. One participant noted: ‘focusing the image was difficult…the photos were blurry'. Others were unsure about the correct positioning and angle for capturing images, resulting in extended time spent on the task.

Opinions on ease of use varied among participants. Some found the app straightforward and user-friendly, with comments such as ‘no challenges at all' and ‘it is easy to use; it is not complicated'; however, others found it time-consuming and suggested that the process could be streamlined.

Suggestions for improvements were focused on enhancing the photo-capture experience and overall user interface. Participants recommended adding a selfie mode or enhanced reality guides to assist users in framing their teeth correctly. The ability to import or adjust photos post-capture was also suggested. Integrating reminders or notifications, syncing with health apps and providing more instructional videos were proposed to improve user engagement and motivation. Key suggestions and corresponding quotes are detailed in Table 4.

**Table 4:** Participant suggestions for the app improvement categorised according to theme

**Theme**	**Participant quotes**
Technical enhancements	Allow photo to be taken in selfie mode to make it easier to frame the teeth
	Implement an AI-guided tool or augmented reality to help position the camera correctly
	Ability to import photos or readjust the photos taken would be helpful
	Using a smaller camera or attachment that can take higher-quality images
User experience	Integrate the app with the Apple health app to track progress and motivate users
	Provide notifications or reminders to encourage regular use of the app
	Include more instructional videos or guidance to help users understand how to use the app effectively
	Prioritise user-friendly design to enhance the overall experience and make it more intuitive
	Using a mirror or providing suggestions on positioning could improve the photo-taking process

## Discussion

This study evaluated the usability and user perceptions of TestMyTeeth, an AI-powered app for at-home dental plaque screening. The findings highlight both the potential of the app in promoting self-monitoring and preventive oral care and the significant usability challenges that could limit its broader adoption. Key issues identified in this study include difficulties in capturing clear intra-oral photographs, user concerns about reliability and accuracy, and the need for professional oversight to enhance trust in AI-generated assessments.

The SUS score of 56.2 falls within the ‘marginal' usability category, below the commonly accepted benchmark of 68. This suggests that while users were able to interact with the app, many encountered frustrations and difficulties in completing tasks efficiently.^10^ However, a SUS score in this range does not indicate outright failure but signals the need for targeted usability improvements to enhance accessibility, functionality and user confidence.

In the broader context of mHealth app usability, similar SUS scores have been reported in AI-powered self-monitoring tools that require precise user input.^9^ Digital health apps that rely on image capture, such as dermatology and ophthalmology self-assessment apps, often receive lower usability ratings when users struggle with lighting, positioning, or clarity of images.^11^^,^^12^ The TestMyTeeth app follows this pattern, as participants found capturing high-quality intra-oral photographs - especially of posterior teeth - to be particularly challenging. Given that AI-driven diagnostics depend heavily on the quality of user-generated input, improving this aspect of the app is crucial for enhancing accuracy and user satisfaction.

This finding aligns with studies on mHealth engagement, which indicate that low usability scores, a lack of experience with mHealth apps and privacy concerns can reduce long-term adherence.^13^ If users find an app difficult to navigate or perceive it as unreliable, they are less likely to integrate it into their daily health routines. Addressing these usability challenges is essential to ensure that TestMyTeeth is both clinically effective and user-friendly.

The most significant usability issue was the difficulty in capturing intra-oral photographs, particularly for the posterior teeth. This limitation affects the core functionality of the app, as high-quality images are necessary for AI-based plaque detection. Previous studies evaluating AI-powered dental health apps have similarly identified image clarity and maintaining user engagement as primary barriers to effective self-monitoring.^9^

Several strategies could enhance the image capturing process:

- Augmented reality guidance - augmented reality guidance has already been implemented in fields such as dental implantology where it has been found to improve accuracy in navigation and placement^14^
- External attachments for stability - devices such as mouth retractors or smartphone mounts could improve lighting conditions and camera stability, reducing variability in image quality
- Haptic feedback and real-time audio guidance - providing tactile or auditory feedback when an image is misaligned or lacks clarity could help users adjust their camera angle and focus.

### Inclusivity and user experience across demographics

One of the key findings of this study is that usability challenges were consistent across all demographic groups. No significant differences were observed based on age, sex, or education level, suggesting that the app's design and functionality need refinement to make it more intuitive and accessible for all users. This is consistent with research on mHealth apps for chronic disease management, which has shown that interface-related barriers affect users regardless of background or experience level.^13^

Improving the usability and guidance system within TestMyTeeth could increase its accessibility and effectiveness, making it a more inclusive tool for diverse populations. Given the high level of interest in self-monitoring, ensuring that the app is user-friendly across different demographics could further enhance its potential for widespread adoption. Many participants appreciated the potential of mHealth apps to promote early detection of oral health issues and potentially reduce the need for unnecessary dental visits. This aligns with a growing body of research, emphasising the shift towards patient-centred care and the role of mHealth apps in empowering users to take charge of their health.^15^

### Concerns about AI reliability and the need for professional oversight

A recurring concern among participants was whether AI-generated assessments could be trusted without professional oversight. Many users hesitated to rely solely on the app's results, fearing that misinterpretations of their oral health status could lead to unnecessary anxiety or delayed treatment, echoing similar concerns raised by other self-diagnosis apps.^16^ This reflects a common challenge in AI-driven healthcare apps, where trust in automated diagnostics is influenced by the absence of human verification and the need for clinical validation, which points to the necessity of integrating professional input into the app.

Similar concerns have been documented in research on AI-assisted dermatology and ophthalmology tools, where users expressed scepticism about self-diagnosis accuracy. The potential for false positives or false negatives in AI-based healthcare apps underscores the need for clinical validation and integration with professional oversight mechanisms.

One way to address these concerns is through teledentistry integration, allowing users to consult with dental professionals if they are unsure about their AI-generated results. This would provide expert verification of AI assessments, reducing the risk of misdiagnosis, and offer personalised recommendations based on both AI analysis and professional input.

### User-driven recommendations for improvement

Participants proposed several key modifications to improve usability. Many users recommended enhancing AI guidance for capturing images, replacing the current static overlay system with real-time positioning assistance. Others suggested adding a selfie mode to help users better position their devices, as well as clearer instructions for capturing images effectively.

These suggestions align with recent research on mHealth usability, which has shown that integrating interactive tutorials, visual aids and adaptive user interfaces significantly improves user engagement and satisfaction.^17^^,^^18^ Participants also highlighted the need for motivational features, such as progress tracking and reminders, which could increase long-term adherence to self-monitoring routines.

### Limitations and future research

While this study provides valuable insights, several limitations must be acknowledged. The convenience sampling method may have introduced selection bias, as participants were likely more technologically literate and more interested in digital health solutions than the general population. Despite efforts to incorporate purposive sampling, the final participant pool was predominantly younger and female, limiting the generalisability of the findings. Additionally, the reliance on self-reported data introduces potential recall bias, as user experiences may not fully reflect actual interactions with the app.

Future studies should aim to increase the diversity of the participant pool, including a broader age range and more male participants, to ensure that findings are more representative of the general population. Further research should also focus on clinical validation, comparing AI-generated plaque detection with professional dental assessments to evaluate its accuracy. Conducting longitudinal studies tracking user engagement and oral health outcomes over time would provide additional insights into the app's effectiveness in real-world settings.

## Conclusion

Users were positive about the potential benefits of including the TestMyTeeth app in their oral care routines, feeling empowered and appreciating the benefits of early detection of pathology. There were significant usability challenges with some of the core functions of the app, most notably, capturing clear intra-oral photographs and navigating the app. These were not related to age, education level, or sex, indicating widespread usability issues. Users also expressed concern about the accuracy of self-assessment without professional guidance, highlighting the need for reliable AI algorithms and confidence-building in data, with clear, actionable feedback from professionals, built into the processes.

## Supplementary Information

Supplementary Information (PDF 405KB)

## Data Availability

Response data are available upon request.
