# Supplementary material for: Evaluating user perceptions and usability of an AI-powered smartphone application for at-home dental plaque screening
Source: Br Dent J. 2025 Jul 11;239(1):46–52. doi: 10.1038/s41415-025-8502-0 (PMC12254032; doi:10.1038/s41415-025-8502-0)
Supplement: Supplementary file 1 — Supplementary Information (PDF 405KB) [file 41415_2025_8502_MOESM1_ESM.pdf]

Appendix 1: Survey form

Part 1: Consent Form

*

Please select 10 options.

- [ ] I confirm that I have read the information sheet dated [18/11/23] version [2] for the above research project.
- [ ] I confirm that I have understood the information sheet dated [18/11/23] version [2] for the above research project and that I have had the opportunity to ask questions and that these have been answered satisfactorily.
- [ ] I understand that my participation is voluntary and I am free to withdraw at any time without giving a reason and without any adverse consequences (e.g. to medical care or legal rights, if relevant). I understand that if I withdraw, information about me that has already been obtained may be kept by Cardiff University.
- [ ] I understand that data collected during the research project may be looked at by individuals from Cardiff University or from regulatory authorities, where it is relevant to my taking part in the research project. I give permission for these individuals to have access to my data.
- [ ] I consent to the processing of my answers to this survey for the purposes explained to me. I understand that such information will be held in accordance with all applicable data protection legislation and in strict confidence, unless disclosure is required by law or professional obligation.
- [ ] I understand who will have access to personal information provided, how the data will be stored and what will happen to the data at the end of the research project.
- [ ] I understand that after the research project, anonymised data may be made publicly available via a data repository and may be used for purposes not related to this research project. I understand that it will not be possible to identify me from this data that is seen and used by other researchers, for ethically approved research projects, on the understanding that confidentiality will be maintained.
- [ ] I understand that anonymised excerpts and/or verbatim quotes from my survey may be used as part of the research publication.
- [ ] I understand how the findings and results of the research project will be written up and published.
- [ ] I agree to take part in this research project.

Part 2: Socio-demographic Background

What is your age? *

- ◯ 16-24
- ◯ 25-34
- ◯ 35-44
- ◯ 45+

What is your Gender? *

- ◯ Female
- ◯ Male
- ◯

What is the highest level of education you have completed/in the process of completing? *

- ◯ Secondary school (GCSE's or equivalent)
- ◯ College/Sixth form (A-level/BTEC or equivalent)
- ◯ University Undergraduate level
- ◯ University Postgraduate level

Part 2:Previous experience of mHealth app use

Do you use medical care-related Apps on your phone (eg MyFitnessPal, Mindspace or others)? *

- ◯ Yes
- ◯ No

Do you use dental care-related Apps on your phone? *

- ◯ Yes
- ◯ No
- ◯ I am not sure
- ◯ If you said yes, please specify below:
- ◯ Other

Have you tried to use a mobile phone to take photos of your teeth or mouth or your child's teeth (before using the Testmyteeth app)? *

- ◯ Yes many times
- ◯ Yes a few times
- ◯ Never

If you replied yes to the question above , on a scale from 1 to 10, how do you rank the difficulty of the experience
(10 being most difficult)?

0	1	2	3	4	5	6	7	8	9	10

Not Difficult — Difficult

Do you think using smartphone teeth pictures is an acceptable idea to examine you (or your child's)teeth and mouth? *

- ◯ Yes
- ◯ No
- ◯ Not sure

Part 3: System Usability Scale tool

Part 4: Feedback on the experience

Have you learnt anything from using this app?

Enter your answer

Are there any adjustments you would like to make to the app?

Enter your answer

What are your opinions on self-monitoring in healthcare?

Enter your answer

Please tell us about the challenges or frustrations you encountered while using Testmyteeth smartphone app:
*

Enter your answer

What suggestions do you have for making it easier to take pictures of teeth?

Enter your answer

Part 4 :Feedback on the experience

How challenging did you find the following statements? *

	**No challenges**	**Minor** (minor, took longer than expected, but able to resolve without help)	**Moderate** (Major delay and/or frustration required verbal guidance from the research team)	**Critical** (Requiring assistance to proceed c demo of the research team)
Navigation of the app interface	○	○	○	○
Following the Testmyteeth app instructions to use	○	○	○	○
Chewing the disclosing agent before taking a photograph	○	○	○	○
Use intraoral camera to take photos of my (or my child's front teeth using the Testmyteeth app	○	○	○	○
Use the intraoral camera to take photos of my (or my child's back teeth using the Testmyteeth app (back teeth)	○	○	○	○
Getting the results from the Testmyteeth app	○	○	○	○
Interpreting the results	○	○	○	○

Part 5: Optional: Summary of results

Thank you for taking part in this study , If you would like a summary of the results , please share your email address below:

Email address:

Enter your answer

Appendix 2: PIS + CF

Dental School Research Ethics Committee

PARTICIPANT INFORMATION SHEET

Introduction

Thank you for considering participation in our research study on the usability of an AI-powered dental screening app. This sheet offers detailed information about the study's purpose and what your involvement entails. Please review it carefully, and don't hesitate to ask questions. We appreciate your consideration in being part of this research**.**

1. **Study title**

   Picture Perfect: Evaluating User Perceptions and the Usability of an AI-Powered Smartphone Application for At-Home Dental Plaque Screening.

2. **Invitation paragraph**

   You are being invited to take part in a research study. Before you decide it is important for you to understand why the research is being done and what it will involve. Please take time to read the following information carefully and discuss it with others if you wish. Please contact Dania Al-zubaidy if you have any questions or require further information. Take time to decide whether or not you wish to take part. Thank you for reading this.

3. **What is the purpose of the study?**

   This study aims to assess the effectiveness and user-friendliness of the Testmyteeth app—an AI-powered smartphone application tailored for at-home dental plaque screening. Leveraging artificial intelligence, the app scrutinises images of users' teeth, providing valuable insights into the presence and distribution of dental plaque.

4. **Why have I been chosen?**

   You have been invited to participate as you are a member of the public who can potentially use this app or similar apps in routine life.

5. **Do I have to take part?**

   Participation in this research is entirely voluntary. The decision to join is entirely up to you. If you choose to participate, please provide your consent by ticking the "I confirm" boxes in part 1 of the survey. If you decide not to participate, simply close the form. You are free to withdraw your consent

at any time without providing a reason, and any data or photographs provided before withdrawal will remain anonymous and cannot be deleted.

6. **What will happen to me if I take part?**

   If you decide to take part in this research you will be asked to download the “TestmyTeeth” app and use a disclosing tablet, following specific instructions to scan your teeth, then complete a short survey which should take no longer than 6 minutes. The data from this survey will be analysed by an undergraduate student and by a member of staff at Cardiff University Dental School. The survey will be open for three months from November 2023 and the research project will be completed in May 2024.

7. **What about confidentiality?**

   Your privacy is of utmost importance to us. Only the research team will have access to the information that you will provide. Therefore, no one can identify you from the images or link you to your submitted photographs on the app. Your identity will remain confidential throughout the study. It's important to note that the data collected is completely anonymous. Your identity will not be disclosed in any reports, publications, or presentations.

8. **What will happen to my personal data?**

   Data will be collected, stored, and processed in-line with GPDR and Cardiff University data guidelines. All data is stored, analysed, and transferred anonymously and confidentially. As part of this study there is no personal data being collected. The photographs you provide will be saved in the app's secure online storage and may be used to train the app artificial intelligence model to recognise various dental diseases and findings. This research does not involve access to your medical records.

9. **What do I have to do?**

   If, after reading this information sheet, you decide you would like to participate in the study, you will be asked to download the Testmyteeth app from Apple/Google stores- here is a link to their website https://www.testmyteeth.com/ . The app will guide you under the “Scan” section and “Guides” in using a disclosing agent provided, following specific instructions to scan your teeth. The plaque disclosing tablet will temporarily stain the surfaces of your mouth containing plaque red and blue (red is newer plaque <12hrs and blue is older plaque >12hrs). The staining can be rinsed out with water and brushing. This process aims to provide insights into dental plaque. Following this experience, you will be asked to fill out an online questionnaire bout the experience of using the app. By submitting the survey you are agreeing for your data to be collected.

10. **Are there any risks?**

There are no apparent disadvantages or risks associated with participating in this study. Taking the photographs is straightforward and non-invasive. There is no harm to subjects in participating in this study or to student progression on their programme of learning.

11. **What will happen to the results of the research study?**

The research project will conclude in May 2024 with the results being published as part of a 4th year undergraduate students FYP. The project may also be submitted for journal publication. Participants will not be identifiable from any of the published information. Summaries of the results will be available upon request.

12. **Who is organising and funding the research?**

This project has received no funding and is being organised as part of a 4th year FYP. The School of Dentistry Research Ethics Committee at Cardiff University has reviewed and approved this study.

13. **Will I be paid for taking part?**

Participation in this study does not come with financial compensation. Your contribution is highly valuable to us.

14. **What are the possible benefits of taking part?**

While there are no direct personal benefits, your participation will contribute to the advancement of dental healthcare technology, potentially benefiting many individuals in the future.

15. **What if there is a problem?**

If you wish to complain or have grounds for concerns about any aspect of the manner in which you have been approached or treated during the course of this research . If you wish to raise a complaint, please notify Dr. Waraf Al-Yaseen (al-yaseenw1@cardiff.ac.uk) in the first instance. If, thereafter, you feel that your complaint has not been handled to your satisfaction, please contact the Chair of the School Research Ethics Committee, Dr. Damian JJ Farnell (farnelld@cardiff.ac.uk).

16. **Contact for Further Information**

If you do have any questions or for further information please contact:

Dania Al-zubaidy – Researcher

Al-zubaidydy@cardiff.ac.uk

Waraf Al-Yaseen – Project supervisor

Al-yaseenw1@cardiff.ac.uk

If you are experiencing difficulties with the app, please contact:

support@testmyteeth.com

or

Al-yaseenw1@cardiff.ac.uk

We appreciate your consideration in participating in this research. Your involvement could contribute to significant advancements in dental care.

research.

THANK YOU FOR PARTICIPATING IN OUR RESEARCH

Consent Form:

Statements	Tick if you are happy to consent
I confirm that I have read the information sheet dated [24/11/23] version [2] for the above research project.	☐
I confirm that I have understood the information sheet dated [24/11/23] version [2] for the above research project that I have had the opportunity to ask questions and that these have been answered satisfactorily.	☐
I understand that my participation is voluntary and I am free to withdraw at any time without giving a reason and without any adverse consequences (e.g. to medical care or legal rights, if relevant). I understand that if I withdraw, information about me that has already been obtained may be kept by Cardiff University.	☐
I understand that data collected during the research project may be looked at by individuals from Cardiff University or from regulatory authorities, where it is relevant to my taking part in the research project. I give permission for these individuals to have access to my data.	☐
I consent to the processing of my answers to this survey for the purposes explained to me. I understand that such information will be held in accordance with all applicable data protection legislation and in strict confidence unless disclosure is required by law or professional obligation.	☐
I understand who will have access to the personal information provided, how the data will be stored and what will happen to the data at the end of the research project.	☐
I understand that after the research project, anonymised data may be made publicly available via a data repository and may be used for purposes not related to this research project. I understand that it will not be possible to identify me from this data that is seen and used by other researchers, for ethically approved research projects, on the understanding that confidentiality will be maintained.	☐
I understand that anonymised excerpts and/or verbatim quotes from my survey may be used as part of the research publication.	☐
I understand how the findings and results of the research project will be written up and published.	☐
I agree to take part in this research project.	☐
